# Are men more likely to experience occupational accidents?

**DOI:** 10.47626/1679-4435-2023-1250

**Published:** 2025-01-07

**Authors:** Claudio José dos Santos Júnior, Frida Marina Fischer

**Affiliations:** 1 School of Public Health, Universidade de São Paulo, São Paulo, SP, Brazil

Dear editors,

We wrote this letter in response to the article “Are men more likely to have serious occupational accidents? Findings of reported cases in Palmas, northern Brazil”,^1^ published in the *Revista Brasileira de Medicina do Trabalho*.

The authors, using data from the Notifiable Injuries Information System (SINAN), analyzed records of serious occupational accidents (OA) that occurred between 2009 and 2019 in Palmas, Tocantins, cross-referencing these data with the economically active population according to sex. It was found that men are 6.2 times more likely to suffer an OA than women.

Inspired by these findings, we decided to reproduce the authors’ method, expanding it, with certain adaptations, to the national level. To do this, we performed the following steps:

1) Through TabNet,^2^ we obtained 2019 OA data reported in SINAN, disaggregated by sex;
2) Through the Brazilian Institute of Geography and Statistics’ Automatic Recovery System (SIDRA/IBGE),^3^ we obtained the number of employed economically active people in Brazil for the fourth quarter of 2019, based on the Continuous National Household Sample Survey, disaggregated by sex;
3) We structured a 2 × 2 contingency table in which the number of OA in each sex was subtracted from the total number of individuals in the employed economically active population, also stratified by sex to compare those who did and did not have an OA; and
4) We calculated the odds ratio to verify the association between sex and the occurrence of OA. Through binomial testing, we verified whether the difference in OA proportions between the sexes was statistically significant. All tests were performed using BioEstat 5.3, with p < 0.05 considered significant.

We found that the distribution of OA between men and women in the economically active population is markedly unequal. Although women represented a significant portion of the economically active population (51.6%) in 2019, men experienced a much higher proportion of OA (79.1% of cases).

The odds ratio of 4.04 (95% confidence interval 3.98-4.09, p < 0.01) confirmed the association between male sex and a higher incidence of OA. The binomial test confirmed that there is a statistically significant difference in the proportion of OA between the sexes in Brazil.

The probability of a man suffering an OA in Brazil in 2019 was approximately 4 times greater than that of a woman under the evaluated conditions. Table 1 summarizes the main results of the statistical analysis.

**Table 1:** Employed economically active population (EEAP) in Brazil and occupational accidents, according to sex

	Total	Male n (%)	Female n (%)
EEAP^*^	169,996	81,714 (48.4)	87,282 (51.6)
Occupational accidents	111,981	88,536 (79.1)	23,445 (20.9)
			
	**OR**	**95%CI**	**p-value**
	4.04	3.98-4.09	< 0.01
			
Binomial test	**z**	**p-value**	**Test power**
	206.33	< 0.01	1

OR = odds ratio; 95%CI = 95% confidence interval.

* 1,000 inhabitants.

Our findings corroborate those of Neves & Fonseca,^1^ i.e., that male sex is associated with a higher occurrence of OA. The results of both studies highlight the urgent need to address sex disparities in the field of occupational safety and health.

Our conclusions are also in line with other national studies that emphasize greater morbidity and mortality due to OA among men.^4-6^

The implications of these findings point to the importance of occupational safety and health policies and practices that are sensitive to sex differences and that address the underlying causes of the predominance of OA in men, ultimately aiming to reduce these events nationwide, especially in this segment of workers.
